# Resource Support for “Mobilization–Participation” in Public Health Emergencies Based on a Complex Network Evolutionary Game

**DOI:** 10.3390/healthcare11101506

**Published:** 2023-05-22

**Authors:** Chenxi Lian, Jida Liu, Jian Wang

**Affiliations:** School of Management, Harbin Institute of Technology, Harbin 150001, China; chenxi_lian@163.com (C.L.); wangjian_hit@yeah.net (J.W.)

**Keywords:** emergency resource, emergency resource network, mobilization–participation, game model, COVID-19

## Abstract

The organized system of emergency resources with the participation of social subjects features a network, which puts forward new requirements for mobilization policies for public health emergencies. Considering the “mobilization–participation” action of the relationship between the government and social resource subjects and revealing the mechanism of governance measures represent the foundation of developing effective mobilization strategies. To analyze the behavior of subjects in an emergency resource network, this study proposes a framework for the emergency actions of government and social resource subjects, as well as clarifies the functions of relational mechanisms and interorganizational learning in decision making. The game model and its rules of evolution in the network were developed by considering the interventions of rewards and penalties. An emergency resource network was constructed on the basis of a response to the COVID-19 epidemic in a city in China, and a simulation of the “mobilization–participation” game was designed and conducted. We propose a path to promote emergency resource actions by analyzing the initial situations and the interventions’ effects. This article suggests that guiding and improving the initial selection of subjects under a certain reward system would be an effective path to facilitate resource support actions during public health emergencies.

## 1. Introduction

Human beings have gradually entered the risk society [1]. The suddenness, abnormality, and complexity of disasters have increased, and extremes have emerged, especially in serious public health emergencies [2]. Diverse organizations and agencies serve as a type of informal insurance to play a sustainable role in emergency relief and disaster recovery [3]. Mobilizing social capital is one of the ways the government and the public sector can promote collective action in response to emergency problems [4,5,6], and this is often found in various emergency planning and preplanning situations. Many regions and organizations attach great importance to mobilizing social efforts to participate in emergency management and try to integrate various social subjects to jointly deal with emergencies by absorbing various resources [5,7,8,9,10]. With this consensus, studies of the mechanisms of action via which governments can mobilize the participation of social subjects [11] have become the theoretical foundation for promoting trends of efficient and positive collective actions in the crisis response, which can be a reference for the improvement of the crisis governance system.

The participation of social organizations In emergency responses is more often associated with resource support, where a large number of social organizations, such as social enterprises and voluntary groups, may undertake the production, transportation, and distribution of material [12]. An emergency resource support system is not only a collection of emergency resources and tasks but also a cross-organizational collection involving multiple subjects [11]. Interorganizational cooperation can enhance the performance of emergency management [12] and mitigate emergency losses, and it is decisive in resource support [13]. Studies have recognized that public–private cooperation [14] is an essential element in the development of emergency resource support systems [15]. Due to the significant differences in management systems and the logic of action between social subjects and government agencies, it is important to further explore the interface and differences between the decision-making mechanisms of governmental mobilization and the subjects’ participation in emergency responses [16,17]. In recent years, Ai et al. studied the location of emergency reserve supplies in a mode of joint government–business collaboration and concluded that such a collaborative model could reduce the total cost of emergency reserve supplies, from which businesses would benefit [18]. Qiu et al. [19] explained the interactions among local emergency management departments, the related enterprises, and senior management departments to research cross-regional collaboration regarding emergency resources and demonstrated the important role of local emergency management departments.

Characterizing the behavioral mechanisms of subjects and the principles of inter-subject interactions is a prevalent research perspective used to explain the formation of cooperation. Some efforts were made by relevant scholars on the cooperative relationship between emergency organizations based on evolutionary game theory [15]. Nan et al. [11] constructed a social network for emergency management (SNEM) and explored the behavioral evolution patterns and the stabilization strategies of a government, social organizations, and the public. Their study showed that a symbiotic SNEM of social organizations and active public response under the path of intense government mobilization is the best strategic combination. Hou et al. [20] proposed the evolutionary game theory to describe the embedded cooperation between a government and nonprofit organizations in the management of geological disasters and explored the factors that influence the cooperative relationship. Du et al. [16] also analyzed the interaction mechanism and influencing factors between government and nonprofit organizations from the perspective of emergency mobilization. Another study explored the cooperative behaviors of a government, enterprises, and the public sector in public health emergencies by developing a tripartite game model, as well as proposed strategies to promote cooperation in response to public health emergencies [20]. It can be seen that evolutionary game theory can be used to clearly and intuitively analyze the cooperative interaction between subjects and the process of decision making [14,15,21], which provides some reference for us to depict the behavioral mechanism of the government’s mobilization and social participation in emergency resource support.

Resource mobilization can be considered one of the important issues in emergency governance [22] during public health emergencies. In some theories, social capital can involve the various resources embedded in social networks [23]. The government resource subjects (GRSs) mobilizing the social resource subjects (SRSs) to participate in the response to public health emergencies have to consider their linked interdependent collaborative relationships to facilitate collective action on issues of common concern, which is also consistent with the concept of network governance theory [24].To a certain extent, the success of emergency relief distribution depends on maintaining an effective, efficient, and timely supply network [25]. In terms of the emergency management, with the use of networks regarded as an acceptable method and perspective, scholars have explored many aspects of networks for discussing the interorganizational relationships in emergency actions, including their formation, evolution and structural characteristics, and effects on cooperation [26,27,28]. Many scholars have also described network attributes by using some common indicators, including network density, network concentration, and cliques, which can be used to measure whether organizations in a network interact intensively or to indicate the performance of the network’s structure [29]. 

In the studies of emergency resource support, Martins et al. [30] used a multi-case approach to study governance mechanisms and their impact on the quality of healthcare supply networks. One study attempted to establish a Social Emergency Resource Monitoring System (SERMS) supported by multilayer recruitment networks including NGOs, nonagreed businesses, and individual donors to maximize the amount of resources collected and minimize logistics costs [31]. In addition, some scholars considered the supply and distribution processes used to build supply chains or supply networks during public crises [32,33,34], where they leaned toward general supply chain studies. Generally, research from the supply chain perspective has mostly examined organizational cooperation in production operations or investigated the efficient allocation of resources, making it difficult to include diversity or the nonproductive nature of organizations, as well as interactions of decision making.

The exploration of emergency management networks is expanding. Recent studies have frequently involved the analysis of the collaboration and cooperation of the subjects in them [25,26,35]. In the field of emergency resources, supply, or logistics, public–private partnerships and collaborations among government, business, and society are desired [14,32]. Meanwhile, interactive decision-making studies based on game theory have been conducted [11,15]. However, as a relatively formal interaction, the “mobilization–participation” decision-making mechanism among the subjects of emergency resources has not yet been reported. In line with the requirements of effective mobilization and participation, it is crucial to further characterize the cooperation model and the behavior of the subjects related to emergency resource support.

In summary, from the two-dimensional action perspective of the mobilization of GRSs and the participation of SRSs, this study explores the characteristics of dynamic interactive decision-making of subjects using an evolutionary game model of an emergency resource network. The initial situations and interventions in the simulation experiment are discussed to explain the law of the dynamic evolution of the emergency resource support network. Our possible contribution lies in the introduction of network relations into the game study of emergency resource mobilization as a complementary work in integrating studies on emergency management networks and mobilization actions, which can help to inspire new thinking in the study of resource mobilization under a relational management perspective. The recommendations made using the simulation analysis can facilitate theoretical references for the development of resource mobilization policies in public health emergencies.

## 2. Materials and Methods

### 2.1. Materials

**Figure 1:**
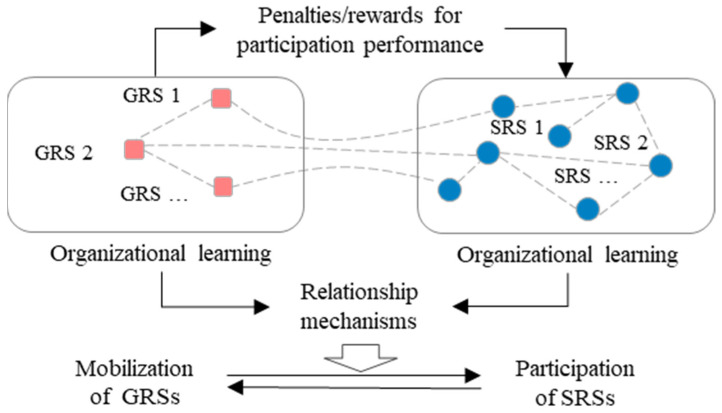
Framework of the “mobilization–participation” mechanism in the emergency resource support network. The red squares represent GRSs and the blue circles represent SRSs.

In resource mobilization theory [36,37,38], organizational mobilization is one of the core elements; it emphasizes the importance of structural factors, such as collective resource availability and the position of the individuals in social networks. The dimension of member mobilization focuses on identifying the mobilizable and potential subjects to be involved in activities through social organizations or interpersonal networks. Participation in collective action is not solely the result of psychological characteristics or predispositions, but also the result of a rational decision-making process in which people weigh the costs and benefits of participation. An emergency resource support system includes both the GRSs and the SRSs, and a network’s structure is formed through communication and cooperation among subjects during an emergency. Therefore, a complex “mobilization–participation” framework can be formed, as shown in Figure 1.

Briefly, in the emergency resource support network, the mobilization and participation actions of the GRSs and SRSs evolve and are updated continuously through the interactive learning of the subjects, finally emerging as the systematic expression of the actions of emergency resource support. 

### 2.2. Methods

In the framework, we focus on the rationality of the network of resource subjects in public health emergencies; however, an elaborate portrayal of the subject’s decision-making mechanisms is needed. To some extent, what we expect is to facilitate collective action in response to public health emergencies, but we have to take into account the differences in the division of labor and the possible differences in the positions of the actors. According to the “mobilization–participation” framework, the decision-making behaviors of GRSs and SRSs are mutually influential and constraining [39]. Game theory is a common approach in decision research [11,15,16], which has obvious advantages in portraying mutually constrained decision behavior (i.e., one subject’s decision is influenced by another subject’s decision, which in turn influences another subject’s decision). Therefore, game theory is adopted to model the interactive decision making of GRSs and SRSs.

#### 2.2.1. Parameters of the Model

In the evolutionary game model of an emergency resource support network, the game’s subjects of GRSs and SRSs can select a positive strategy or a negative strategy. The relevant parameters for the strategy selection are as follows: the probabilities of GRSs adopting a positive mobilization strategy and a negative mobilization strategy are r and 1 − r, respectively (0 < r < 1). The probabilities of SRSs adopting a positive participation strategy and a negative participation strategy are m and 1 − m, respectively (0 < m < 1).

The parameters of costs and returns generated by their differentiated strategy selection are as follows: the routine operating returns of SRSs are π_e_ (π_e_ > 0), and their positive participation in emergency resource support will incur additional costs c_e_ (π_e_ > c_e_). For GRSs, social effects and credibility are important criteria that influence their behaviors. Therefore, when the GRSs mobilize positively, they gain enhanced credibility, denoted as π_g_ (π_g_ > 0), and pay the extra organizational cost c_g_ (c_g_ > 0). When they choose a negative mobilization strategy, they do not receive additional benefits or pay additional mobilization costs, but they lose their authority and credibility in the eyes of the social resource subjects, denoted as c_v_ (c_v_ > 0). 

The parameters related to the intervention involve rules for punishment and reward. The positive GRSs implement intervention measures to induce the positive participation of the SRSs. If both of them take positive actions, the SRSs receive appropriate recognition and reward, which are recorded as additional gains γ (γ > 0). When an SRS adopts a negative strategy, the GRSs can impose a penalty δ (δ > 0), and the overall emergency gains can be compensated for with the probability β (β > 0); that is, the gains of positive government mobilization increase by βδ. With a negative mobilization strategy, GRSs cannot influence the decisions of SRSs because the GRSs do not observe the actions of the SRSs in a timely manner. However, when the SRSs also adopt the same negative participation strategy, the joint negative action will cause public dissatisfaction with the government’s execution, denoted as c_s_ (c_s_ > 0).

#### 2.2.2. Gaming Strategies

According to the previous description, the payoffs of the GRSs and SRSs in the emergency resource support system can be put forward. The payoff matrix of the “mobilization–participation” game system is shown in Table 1.

Without considering the network’s relationships, according to the payoff matrix of the “mobilization–participation” game system, the expected gain of the SRSs is given as

(1) $$H_{e}=mH_{e1}+(1-m)H_{e2},$$

where $H_{e1}=r\left(\pi_{e}-c_{e}+\gamma \right)+(1-r)(\pi_{e}-c_{e})$ is the expected gain of the social resource subject from selecting the positive participation strategy, and $H_{e2}=r\left(\pi_{e}-\delta \right)+(1-r)\pi_{e}$ is the expected gain of the social resource subject from selecting the negative participation strategy.

The expected gain of the GRSs is given as follows:

(2) $$H_{g}=rH_{g1}+(1-r)H_{g2},$$

where $H_{g1}=m\left(\pi_{g}-c_{g}\right)+(1-m)(\pi_{g}-c_{g}+\beta \delta )$ is the expected gain of the GRSs from selecting the positive mobilization, and $H_{g2}=m\left(-c_{v}\right)+(1-m)(-c_{v}-c_{s})$ is the expected gain of the GRSs from selecting the negative mobilization strategy.

Therefore, the dynamic equations of replication are as follows:

(3) $$\frac{dm}{dt}=m(H_{e1}-H_{e})=m(1-m)(r\gamma +r\delta -c_{e}),$$

(4) $$\frac{dr}{dt}=r(H_{g1}-H_{g})=r(1-r)(\pi_{g}-c_{g}+\beta \delta +c_{s}+c_{v}-mc_{s}-m\beta \delta ).$$

Using $\frac{dm}{dt}=0$ and $\frac{dr}{dt}=0$, five possible stable points of (0, 0), (0, 1), (1, 0), (1, 1), and (m^*^, r^*^) were obtained; $m^{*}=\frac{\pi_{g}-c_{g}+\beta \delta +c_{v}+c_{s}}{\beta \delta +c_{s}}$ and $r^{*}=\frac{c_{e}}{I+\delta }$ when $0\leq c_{e}\leq \gamma +\delta$ and $\pi_{g}+c_{e}\leq c_{g}\leq \pi_{g}+\beta \delta +c_{v}+c_{s}$. The Jacobian matrix was constructed from the equation of the replication dynamics, and Det (*J*) and Tr (*J*) were calculated to analyze the stability.

According to Table 2, it can be seen that the stable points under certain conditions are (0, 1) and (1, 1). It can be tentatively suggested that it is likely that the GRSs will need to take positive actions, but the positive participation of the SRSs requires conditions. According to the results of the stable points, the payoff matrix of the “mobilization–participation” game system for emergency resource support is considered to be quite reasonable, given the extraordinary risk and the tendency to save resources in an emergency.

We briefly described the evolutionary trends of the game in the absence of the network’s relationships above. However, the participation of SRSs brings a large number of relationships into the governance system of emergency resource support. Factors such as organizational learning and relational mechanisms become important processes and need to be brought into the model.

#### 2.2.3. Evolutionary Rules

The “mobilization–participation” game of emergency resource support follows the principle of evolutionary games with complex networks [40,41]. It can be described briefly. First, since each edge connects a pair of subjects, each subject needs to conduct games with its neighbors in each round and make a strategic choice. After each round of the game, the payoffs of this round can be calculated. Each subject can learn and decide in the next round. That is, each subject can compare its gain with that of its neighbor to consider whether to adopt its neighbor’s strategy in the next round. Finally, after several rounds of the game, the subjects no longer change their strategies, and the whole system reaches a relatively stable state.

According to the rule above, the probability that the subjects will be selected as the object to be learned is shown in Equation (5).

(5) $$B_{j}=\frac{\exp((u_{j}(t)+\alpha_{j})/\lambda )}{\sum_{j}^{J}\exp((u_{j}(t)+\alpha_{j})/\lambda )},$$

where *B_j_* represents the probability that subject *j* will become the learning object, $u_{i}(t)$ and $u_{j}(t)$ are the gains of subjects *i* and *j* in the previous round, respectively, *a_j_* is an adjusting weight that can change the propensity for high gains, and *λ* is an information cost parameter. Due to the constraints of their information processing abilities and rational negligence [42], subjects can only select objects for empirical learning from their neighbors.

The update rule [43] for each subject’s strategy is shown in Equation (6).

(6) $$\omega_{\left(s_{i}\to s_{j}\right)}=\frac{1}{1+\exp[(u_{i}(t)-u_{j}(t))/\xi ]},$$

where $\omega_{\left(s_{i}\to s_{j}\right)}$ is the probability that subject *i* adopts subject *j*’s strategy, and *ξ* denotes the noise parameter. If ξ→0, subject *i* adopts the strategy of subject *j* only when $u_{i}(t)<u_{j}(t)$. If ξ→∞, subject *i* will make a random choice about whether to change the current strategy.

## 3. Results

### 3.1. Simulation Design

#### 3.1.1. Simulation Context

**Figure 2:**
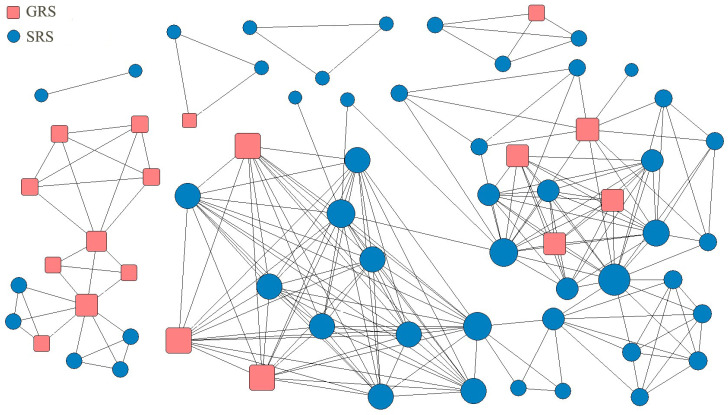
Structure of the emergency resource support network.

During the COVID-19 pandemic, the availability of resources in social and economic activities was greatly affected [44]. The cooperation between GRSs and SRSs regarding providing resource support formed representative interorganizational relationships in response to this emergency. In our study, the fluctuations in the local phase of the epidemic that occurred from February to April 2021 in City S of Province H in China were selected as the case context. We used “epidemic” and “supplies” as keywords to collect news and information related to the development of emergency resource support activities. By extracting the correlations between emergency organizations from the perspective of the “mobilization–participation” concept, the emergency resource support network was constructed, as shown in Figure 2. This network had the topological structure of the evolutionary game of emergency resource support. It should be noted that the emergency resource support network included interorganizational cooperative relationships between GRSs and SRSs, as well as same-type cooperative relationships in GRSs or SRSs.

#### 3.1.2. Simulation Settings

Before the simulation experiment was conducted, the model’s parameters needed to be set according to the practical case and the network’s structure. According to the characteristics of the organizations in the case, the subjects were divided into two categories, namely, 19 GRSs and 49 SRSs. For the calculations, the parameters related to SRSs were set to *π_e_* = 8 and *c_e_* = 2, and the parameters related to GRSs were *π_g_* = 3, *c_g_* = 5, *c_v_* = 1, *c_s_* = 2, and *β* = 0.3. Parameters such as the information cost and noise were not involved in this focused discussion and were set to *λ* = 1, *ξ* = 1, and *α_j_* = 0.

**Figure 3:**
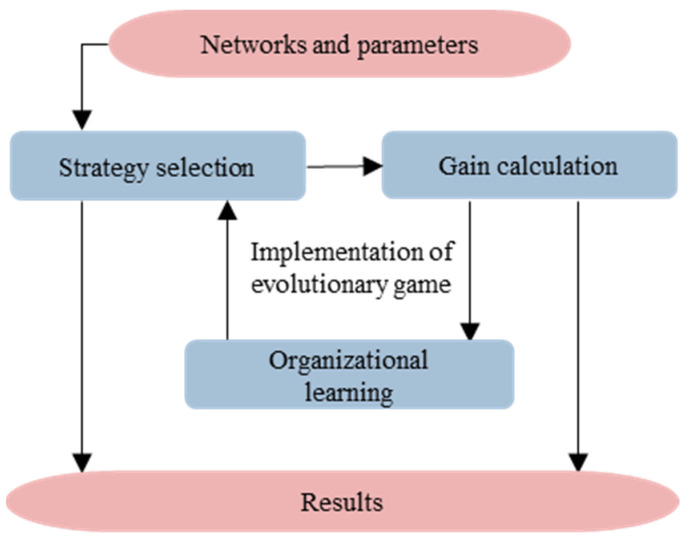
Flow of the evolution of the simulation network game.

The addition of relationship mechanisms and organizational learning enhanced the complexity of the game’s evolution. While the principle of agent-based modeling can facilitate simulations of the complex decision-making behavior of subjects, it can also clearly show the decision-making process of micro-subjects, and the results emerge at the system level. Therefore, Netlogo [45] software was used to import the structure and basic parameters of the network, the “mobilization–participation” strategy was selected, and then the subject’s gain for a given round was calculated. Subjects observed and learned the decisions and gains of their neighbors and then decided which strategy to adopt, thus achieving round-by-round evolution of the game. The flow of the simulation experiment is shown in Figure 3. In addition, due to the presence of a stochastic process in organizational learning, the simulation experiments were repeated 20 times with each set of settings to reduce the effect of random variables on the results.

**Figure 4:**
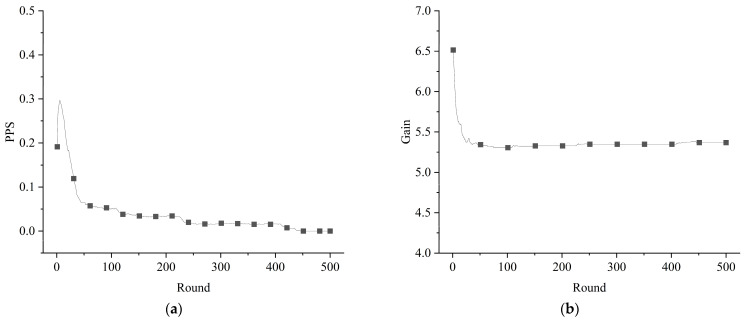
Evolution of the game system without interventions: (**a**) evolution of the proportion of positive strategies; (**b**) evolution of the average gain of the subjects.

In accordance with the requirements of the established model, the evolutionary game system of the emergency resource support network could be simulated by giving values for the parameters. With the reward and penalty parameters set as 0, we took the probability of GRSs choosing the positive mobilization strategy in the initial situation to be *r*_0_ = 0.4 and the probability of SRSs choosing the positive participation strategy in the initial situation to be *m*_0_ = 0.1. After the simulation process, the evolutionary process of the overall proportion of the positive strategy (PPS) and the average gain of the subjects in the emergency resource support network were obtained, as shown in Figure 4. It can be seen that, in the absence of rewards and penalties, the evolution of the game system of the emergency resource support network stabilized at the level of PPS = 0. In other words, the situation of collective action in which the GRSs and SRSs were always positively mobilizing and participating was hard to achieve without an intervention.

### 3.2. Analysis

#### 3.2.1. Initial Conditions

**Figure 5:**
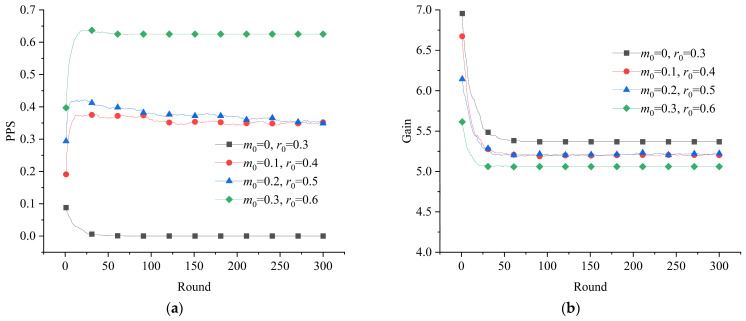
Effect of the initial situation on the evolution of the game system: (**a**) evolution of the proportion of positive strategies; (**b**) evolution of the average gain of the subjects.

To analyze the role of the subjects initially taking positive actions in the emergency governance system, four differentiated initial proportions of positive strategies were set, and the results of the simulation are shown in Figure 5. Because social cooperation seldom forms naturally, an award mechanism with *γ* = 0.6 was set to promote the effectiveness of emergency resource support actions. 

The results showed that an increase in the initial proportion of positive strategies helped to increase the number of positive “mobilization–participation” actions toward emergency resource support at a system level. According to the results of the simulation experiment, when the initial proportion of positive strategies was low (*m*_0_ = 0, *r*_0_ = 0.3), the final proportion of positive strategies in the system tended to be 0. Meanwhile, the situation improved when the initial proportion of positive strategies increased. In particular, when the proportion of positive GRSs was 0.6 and the proportion of positive SRSs was 0.3, there was a significant improvement in the evolutionary results. Although the average gain of the subjects was not high in this situation, it still contributed to a certain degree of positive action in emergency resource support. The initial positive actions reflect the emergency preparedness and the accumulation of emergency social capital of the GRSs and SRSs. The organizations that can commit to taking emergency resource support actions at first are usually government departments with emergency management tasks, reserve agencies, charitable relief organizations, and social enterprises that have experience in participating in emergency operations, etc. The simulation results indicated that strengthening a series of preconstructed emergency plans, emergency reserves, and social emergency preparedness that can improve the subjects’ capacity for rapid response is an urgent requirement for improving the performance of the resource support system during a public health emergency.

#### 3.2.2. Interventions

**Figure 6:**
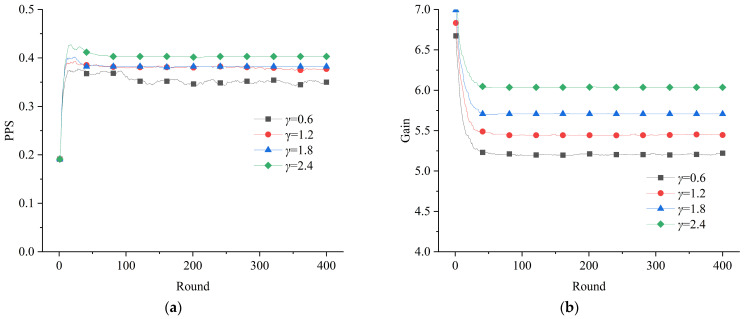
Effect of rewards on the evolution of the game system: (**a**) evolution of the proportion of positive strategies; (**b**) evolution of the average gain of the subjects.

**Figure 7:**
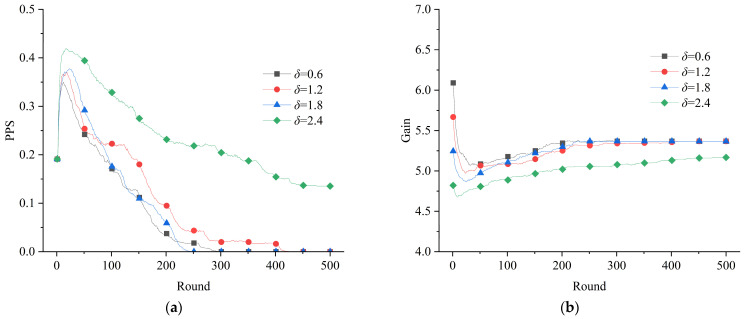
Effect of penalties on the evolution of the game system: (**a**) evolution of the proportion of positive strategies; (**b**) evolution of the average gain of the subjects.

According to the previous analysis, the development of interventions is one of the aspects necessary to promote the construction of emergency resource support systems. To analyze the role of the rewards and punishments, considering the initial situation parameters of *r*_0_ = 0.1 and *m*_0_ = 0.4, different levels of reward and penalty were set to observe the evolution of the emergency resource support network. The results are depicted in Figure 6 and Figure 7. These results show that the effects of rewards and penalties on the “mobilization–participation” actions of emergency resource support were different, in which rewards were more likely to promote positive actions. By comparing Figure 4 and Figure 6, it can be seen that imposing rewards was significant for consistently increasing the gains of emergency resource subjects. The effect of rewards on promoting positive actions was also obvious. However, varying the level of the rewards did not make a large difference in the final results for the proportion of positive strategies. As shown in Figure 4 and Figure 7, the final results of the game’s evolution seldom tended to positive actions in the absence of penalties and with low penalties. Only when the penalty parameter *δ* = 2.4 did the positive actions of “mobilization–participation” start to stabilize at a certain value. In terms of the gain indicator, the change in the average gain of the subjects was more complicated because of the subsidies *β* that the government applied. Synthetically, rewards are more likely to promote the positive actions of resource subjects during a public health emergency, while penalties require a high level of enforcement to work.

#### 3.2.3. Comprehensive Perspective 

**Figure 8:**
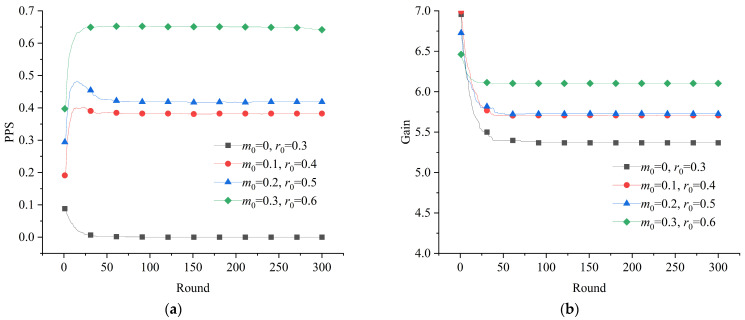
Evolution of the game system with *γ* = 1.8: (**a**) evolution of the proportion of positive strategies; (**b**) evolution of the average gain of the subjects.

It is necessary to analyze the initial conditions and interventions from a comprehensive perspective to identify effective paths to promote positive actions. Since rewards are relatively more likely to promote positive strategies, the system’s evolution was observed under the condition of *γ* = 1.8, with different initial proportions of positive strategies. The results are shown in Figure 8. 

The simulation’s results show that the effects of initial conditions and rewards were superimposable. Specifically, at a certain reward level, the increase in the initial proportion of positive strategy could expand the measured effect. On the one hand, the results of evolutionary stabilization appear to show a dilemma when the initial proportion of positive strategies was low, i.e., *m*_0_ = 0 and *r*_0_ = 0.3, while the results increased sequentially when the proportion of initial positive strategies increased, as shown in Figure 8a. This is consistent with the evolutionary trend with *γ* = 0.6 for the different initial conditions in Figure 5a. On the other hand, compared with the modest difference in the effects of different reward levels exhibited in Figure 6a and Figure 8a, an increase in the initial proportion of positive strategies drove a significant increase in the effect of rewards. Overall, increasing the initial proportion of positive strategies at a certain reward level was effective in improving the final effect of the system. Conversely, increasing the reward level under certain initial conditions had little impact on improving the results. That is, the former was the most effective path to improve the system’s performance during a public health emergency.

## 4. Discussion

Resource support in emergency scenarios requires the positive participation of social subjects. The mobilization of the government and its agencies is an inherent part of building emergency response capacity, and the effect of mobilization might affect the organizational structure of the emergency response [4,11]. On the basis of the “mobilization–participation” behavior mechanism in the organizational network of emergency resource support, an evolutionary game model of the network was developed. A network was constructed, and a simulation experiment was conducted in the context of a Chinese city responding to COVID-19. It was found that the initial proportion of positive strategies in the simulation experiment was a positive element for promoting favorable actions at the system level. Interventions were necessary, although the effects of rewards and punishments were not the same. The results of our simulation of these two aspects further explain the overall thinking on the factors for improving the “mobilization–participation” of emergency resource support, which is a valuable reference for developing emergency policies.

In this study, we defined the interaction between GRSs and SRSs in the resource network as “mobilization–participation”. Then, we proposed the rules of interactive decision making using game theory and conducted a case simulation on a city’s public health emergency response. Although direct generalization of the results may be difficult in the case of our simulation, the differences in initial conditions and interventions effects shown by the results are informative for the governance of the public health emergency in the case, which imply the need for decision-making interactions and relational considerations. For example, in this case, when more subjects took positive “mobilization–participation” actions at the beginning, it was easier to create an atmosphere of positive responses, which influenced the choices of other subjects. This result is understandable from the point of view of organizational learning. In terms of the intervention’s effect, we built on previous studies [16,21,46] to explain the possible differences in the effects of reward and penalty measures in more detail. This may help to remind emergency resource policymakers to pay sufficient attention to the rational configuration of rewards and penalties.

In our study, network relationships were introduced into the game analysis of resource mobilization in public health emergencies, which contributes to enriching the study of network governance in emergency management. The simulation-based dynamic analysis also provides a flexible way of examining the practical problems and policy formulation of resource mobilization in public health emergencies. Using the “mobilization–participation” game from the perspective of resource support networks during a public health emergency, we unveiled some helpful findings for improving policies, but there were still shortcomings to this study. Practically speaking, the various aspects of a public health emergency are considerably complex. This complexity may arise from at least three aspects: subjects, relations, and decision mechanisms. With the deepening of typological research at the subject level, the subject can also be continuously subdivided and functionalized in some qualitative and detailed explorations. Both strong and weak relationships exist in social networks, and their roles in resource networks may also be differentiated. In fact, both of the above can influence the decision-making mechanisms of subjects under network governance theory. We consider that these three aspects may be directions for continuous improvement of research under this framework and help to promote the theory and practice of network governance in public health emergencies. 

## 5. Conclusions

Social participation provides an indispensable complement to emergency responses in large-scale public emergencies. As the emergency resource support network is composed of GRSs and SRSs, it is necessary to consider the learning of subjects to make complex decisions in reality. On this basis, the “mobilization–participation” mechanism and an evolutionary game model in the emergency resource support network were proposed, with a network constructed using a case in the context of the COVID-19 epidemic in China. The impacts of the initial conditions and interventions on the selection of strategies by the subjects were analyzed by conducting agent-based simulation experiments, and the best path to enhance the positive actions of emergency resource support was proposed.

According to the results of the case study, the improvement can be made in the following aspects.

(1) Comprehensively strengthen the construction of resource reserves and improve the level of preparedness of the whole society for public health emergencies. 

The results of this case study illustrated that a higher initial proportion of positive strategies was associated with better results at the system level. Meanwhile, the description of the effective paths also indicated the significance of increasing and improving the initial proportion of positive strategies. While the initial proportion of positive strategies is an essential reflection of the positive actions and resources invested in the early stages of the emergency response to a certain extent, to achieve a rapid response and the positive engagement of emergency resource subjects at an early stage, it is necessary to strengthen emergency resource preparedness and enhance the capacity for response to improve the effectiveness of the initial actions. On the one hand, it is necessary to accumulate practical experience, strengthen the effects of social mobilization, and expand the scope of participation and the willingness of subjects to participate. On the other hand, it is advisable to enhance the education and training of the subjects, as well as to improve the mechanism of normalized emergency preparedness and resource reserve management to enhance the response rate and emergency disposition capacity of society. 

(2) Because of the complexity of the risk in responding to public health emergencies, various resource action interventions should be scientifically developed. 

Rewards and penalties are common interventions in policy systems, and our study explained the different effects of rewards and penalties in the “mobilization–participation” mechanism of emergency resource support. Actually, reward measures in emergency governance are also a cost of national government expenditure to a certain extent, and very high rewards would not have great effects. Therefore, the establishment of a reward system with reasonable intensity and scope according to the local conditions and circumstances is essential to mobilize emergency resources. In addition, given the effectiveness of penalty measures and the pressure of unexpected events, the design of penalty measures should be kept prudent.

(3) The relationships between subjects can be strengthened in order to improve the structure of the governance network for resource support during public health emergencies. 

In the network, relationships provide the essential channels for obtaining information for making empirical decisions and are important factors influencing the formation of the atmosphere of collective action. Moreover, the formation of structural relationships contributes significantly to expanding the organizational system of emergency resource support. Therefore, it is imperative to guide and strengthen the construction of interorganizational relationships in the emergency resource system to improve the governance structure and enhance operational capabilities. Building relationships can be carried out in various ways. For example, policy documents, such as emergency plans, can be continuously improved to clarify the command-and-collaboration relationships between organizations in rapid emergency operations. For the social resource subjects, a cooperative relationship can be generated through the supply chain, the cross-regional industrial complementarity, and the government’s guidance.

## Figures and Tables

**Table 1:** Payoff matrix of the “mobilization–participation” game system for emergency resource support.

Game Systems		Government Resource Subjects	
		Positive Mobilization *r*	Negative Mobilization 1 − *r*
Social resource subjects	Positive participation *m*	$\pi_{e}-c_{e}+\gamma$ $\pi_{g}-c_{g}$	$\pi_{e}-c_{e}$ $-c_{v}$
	Negative participation 1 − *m*	$\pi_{e}-\delta$ $\pi_{g}-c_{g}+\beta \delta$	$\pi_{e}$ $-c_{v}-c_{s}$

**Table 2:** Determination of the stable points of the game system.

Points	Det (*J*)	Tr (*J*)	Stability	Conditions
(0, 0)	$-c_{e}(\pi_{g}-c_{g}+\beta \delta +c_{s}+c_{v})$	$-c_{e}+\pi_{g}-c_{g}+\beta \delta +c_{s}+c_{v}$	N	-
(0, 1)	$\left(c_{e}-\gamma -\delta )(\pi_{g}-c_{g}+\beta \delta +c_{v}+c_{s}\right)$	$\gamma +\delta -c_{e}-(\pi_{g}-c_{g}+\beta \delta +c_{v}+c_{s})$	Y	$\gamma +\delta <c_{e}$
(1, 0)	$c_{e}(\pi_{g}-c_{g}+c_{v})$	$c_{e}+\pi_{g}-c_{g}+c_{v}$	N	-
(1, 1)	$\left(\gamma +\delta -c_{e})(\pi_{g}-c_{g}+c_{v}\right)$	$-(\gamma +\delta -c_{e})-(\pi_{g}-c_{g}+c_{v})$	Y	$\gamma +\delta >c_{e}$
(m*, r*)	$\frac{c_{e}(\rho_{g}-c_{g}+\beta \delta +c_{v}+c_{s})(\pi_{g}-c_{g}+c_{v})(c_{e}-\gamma -\delta )}{\left(\beta \delta +c_{s})(\gamma +\delta \right)}$	0	N	-

## Data Availability

Data sharing is not applicable.
